# The association between socioeconomic deprivation and secondary school students’ health: findings from a latent class analysis of a national adolescent health survey

**DOI:** 10.1186/s12939-016-0398-5

**Published:** 2016-07-16

**Authors:** Simon Denny, Sonia Lewycka, Jennifer Utter, Theresa Fleming, Roshini Peiris-John, Janie Sheridan, Fiona Rossen, Donna Wynd, Tasileta Teevale, Pat Bullen, Terryann Clark

**Affiliations:** Department of Paediatrics: Child and Youth Health, Faculty of Medical and Health Sciences, The University of Auckland, Private Bag 92019, Auckland, 1142 New Zealand; Section of Epidemiology and Biostatistics, Faculty of Medical and Health Sciences, The University of Auckland, Auckland, New Zealand; School of Pharmacy, Faculty of Medical and Health Sciences, The University of Auckland, Auckland, New Zealand; Department of Social & Community Health, Faculty of Medical and Health Sciences, The University of Auckland, Auckland, New Zealand; Child and Youth Health Team, Auckland District Health Board, Auckland, New Zealand; Academic Division, University of Otago, Auckland, New Zealand; School of Learning Development and Professional Practice, The University of Auckland, Auckland, New Zealand; School of Nursing, Faculty of Medical and Health Sciences, The University of Auckland, Auckland, New Zealand

**Keywords:** Socioeconomic deprivation, Poverty, Adolescence, Mental health, Obesity, Cigarette use, Latent class analysis, Multilevel modelling

## Abstract

**Background:**

The aims of this study were to examine indicators of socioeconomic deprivation among secondary school students and to determine associations between household poverty, neighbourhood deprivation and health indicators.

**Methods:**

Data were from a nationally representative sample of 8500 secondary school students in New Zealand who participated in a health survey in 2012. Latent class analyses were used to group students by household poverty based on nine indicators of household socioeconomic deprivation: no car; no phone; no computer; their parent/s worry about not having enough money for food; more than two people sharing a bedroom; no holidays with their families; moving home more than twice that year; garages or living rooms used as bedrooms; and, no parent at home with employment. Multilevel generalized linear models were used to estimate the cross-level interaction between household poverty and neighbourhood deprivation with depressive symptoms, cigarette smoking and overweight/ obesity.

**Results:**

Three groups of students were identified: 80 % of students had low levels of household poverty across all indicators; 15 % experienced moderate poverty; and 5 % experienced high levels of poverty. Depressive symptoms and cigarette smoking were 2–3 times higher in the poverty groups compared to student’s not experiencing poverty. There were also higher rates of overweight/ obesity among students in the poverty groups compared to students not experiencing poverty, but once covariates were accounted for the relationship was less clear. Of note, students experiencing poverty and living in affluent neighbourhoods reported higher levels of depressive symptoms and higher rates of cigarette smoking than students experiencing poverty and living in low socioeconomic neighbourhoods. This cross-level interaction was not seen for overweight/ obesity.

**Conclusions:**

Measures of household socioeconomic deprivation among young people should not be combined with neighbourhood measures of socioeconomic deprivation due to non-linear relationships with health and behaviour indicators. Policies are needed that address household poverty alongside efforts to reduce socioeconomic inequalities in neighbourhoods.

## Background

Socioeconomic deprivation measures are widely used in health research in order to study the effects of deprivation and poverty and to control for possible confounding by socioeconomic factors [1–4]. While there has been an interest in studies exploring the role of socioeconomic status and health, there are few studies exploring issues to do with the measurement of socioeconomic status, especially in relation to the measurement of poverty among adolescents [5–7]. This is partly due to theoretical and conceptual issues as there is no general consensus of what constitutes socioeconomic deprivation or poverty. There are also difficulties in establishing indicators of poverty that are valid for use in self-report adolescent health surveys.

In most economically developed nations, poverty is defined in relative terms as the “exclusion from the minimum acceptable way of life in one’s own society because of inadequate resources” [8]. Low income is the most commonly used indirect measure of poverty and it is often used as a sole indicator. However, there are problems with relying on income as a single measure of poverty [9]. It is difficult to define a minimum income level below which poverty results. This is compounded by the fact that there is substantial mismatch between self-reported income and more direct measures of poverty, such as current living standards [8].

Measuring socioeconomic deprivation among adolescents brings further difficulties as they often have no income or employment, are still dependent on their families and are yet to complete their education. One approach estimates the socioeconomic position(s) of adult(s) in the home by traditional measures of socioeconomic status such as household income, highest completed education or occupation and applies this to young people and children living in the home. However, for surveys which collect information solely from young people, their knowledge of their parents’ income or education levels is often inaccurate [10]. In response to these concerns, Currie et al. developed the family affluence scale to measure socioeconomic status among younger adolescents by asking adolescents directly about their family’s car ownership, whether they have their own bedroom, number of family holidays in the past year and number of computers in their home. The current study expands on the study by Currie et al, by analysing nine indicators of socioeconomic deprivation taken from a national survey of young people.

The move towards using hardship indicators of poverty, such as the family affluence scale, poses problems in terms of how to combine multiple indicators. A simple composite or linear combination of indicators ignores the multidimensional nature of these indicators and assumes selected indicators are measured without error [11]. A better approach is to use latent class analyses (LCA) that combines multiple indicators of deprivation to identify groups within a population experiencing poverty. A latent class analysis assumes the axiom of local independence whereby an underlying latent categorical variable explains the correlations between multiple indicators [12]. While this has been used previously to classify socioeconomically deprived individuals it has not been employed with an adolescent sample using self-reported data [13–15].

Using data from a large nationally representative youth health survey, this paper examines indicators of socioeconomic deprivation and how these indicators vary by demographic characteristics of adolescents. We identify adolescents experiencing household poverty, using latent class analysis, and examine the relationship with a well-established measure of neighbourhood deprivation in New Zealand, the New Zealand 2013 Deprivation Index [16]. We then examine the relationship between adolescents experiencing household poverty, neighbourhood deprivation and health indicators using cross-level interaction multilevel models.

## Methods

A two-stage cluster design was used to obtain a nationally representative sample of New Zealand secondary school students. Sample size calculations for this survey aimed to give reasonable prevalence estimates of health indicators among the 4 main ethnic groups in New Zealand [17]. In 2012 there were 493 composite or secondary schools with year 9 students (average age 13 years) and above. Excluding schools with fewer than 50 students and Kura Kaupapa Māori schools (indigenous immersion schools) there were 397 eligible schools from which 125 were randomly selected and invited to participate. Of these, 91 schools (73 %) took part in the survey. In each participating school we randomly selected 20 % of all year 9 to 13 students (aged 13 to 17 years) from the school roll and invited them to take part. In total, 12,503 students from 91 consenting schools were randomly selected and invited to participate in the survey. Of these, 8500 (68 %) took part. Reasons for students not participating included students declining (20.3 %), students being absent or no longer at school (24 %) or being involved in other school activities (10.8 %). For many non-participating students there was no information available.

School principals gave consent for their own school to take part. Information on the survey was sent to each school for distribution to parents and students. Parents were able to opt to have their child withdrawn from the study. Each student gave their own consent to participate at the beginning of the survey. All students in each school were eligible to participate; the only exclusion criterion was the inability to participate in the survey (4.5 % of students were excluded due disability, language or reading ability). Ethical approval was obtained from the University of Auckland Human Participants Ethics Committee (2011/206). The survey was carried out from March through November in 2012 and administered using audio computer-assisted self-interviewing on internet tablets [18].

Indicators of household socioeconomic deprivation were developed from the New Zealand census, previous questionnaires and included items from the Health Behaviour in School-Aged Children (HBSC) family affluence scale [6]. Cut-points for each indicator were determined through discussion with researchers and previous research [19–21]. Household socioeconomic deprivation indicators and health indicators are described in Table 1. Age, sex and ethnicity were determined by self-report. Ethnicity was assessed using the standard New Zealand Census ethnicity question where participants can select all of the ethnic groups with which they identify. Approximately 42 % of students identified with more than one ethnic group. To facilitate statistical analyses, discrete ethnic groups were created using the New Zealand census prioritisation method by assigning students to one ethnic group in the following order; Māori (20 %), Pacific (14.1 %), Asian (12.4 %), other ethnic groups (6 %) and New Zealand European (47.4 %).

**Table 1:** Description of outcome variables and socioeconomic deprivation measures

Health/ Behaviour Measures	Description of Measures
Depressive symptoms (10 items)	Students’ depressive symptoms were assessed by the Reynolds Adolescent Depression Scale – Short Form (Mean 19.56, SE 0.07, Range 10–40, Cronbach’s alpha 0.90). This is a well validated 10-item questionnaire that measures depressive symptoms among adolescents. Based on previous analyses, students with a score greater than 28 were classified as having clinically significant depressive symptoms [34, 35].
Overweight/Obese	Anthropometric measures were taken by trained research staff following standardized procedures and protocols. Height was measured using a portable stadiometer (Seca model 214, Seca, Hamburg, Germany) to the nearest 0.1 cm. Weight was measured using digital scales (Health-o-meter model 349KLX, Health-o-meter, Bridgeview, IL) to 0.1 kg. Body mass index was calculated by dividing weight (kg) by height (m) squared. Students were classified as overweight/obese based on age and sex-specific BMI definitions as recommended by the International Obesity TaskForce [36].
Weekly cigarette smoking	Weekly cigarette smoking was assessed by two questions: “Have you ever smoked a whole cigarette?” with response options “yes”; “no”; and “How often do you smoke cigarettes now?” with response options: “Never – I don’t smoke now”; “Occasionally”; “Once or twice a month”; “Once or twice a week”; “Most days”; and, “Daily”. Students who reported smoking weekly or more often were classified as weekly cigarette smoking”.
Household socioeconomic deprivation measures	
Household goods: car/ telephone/ computer	To assess household goods, students were asked: “In your home how many of the following things are there?”, with response options:”A car that goes”;”A telephone that works”; “A computer/ laptop” with response options “None”; “One”; “Two”; “Three or more”. Students who reported no car, no telephone or no computer were classified into the “No car’, “No telephone” and “No computer” groups respectively.
Parents worry about having enough money for food	Students were asked “Do your parents, or the people who act as your parents, every worry about not having enough money to buy food?” with the response options “Never”, “Occasionally”, “Sometimes”, “Often” and “All the time”. Students who responded “Often” and “All the time” were classified as “Parents worry about having enough money for food”.
More than 2 people per bedroom	Overcrowding was calculated from the responses to two questions: “How many bedrooms are there where you live?” with response options “None”, “1”, “2”…”10”, “more than 10” and “How many people, including you, usually live in your main or only home?”. Students who reported more than two people per bedroom were classified as “More than 2 people per bedroom”.
No family holiday in last 12 months	Information on Family holidays was obtained by asking the question: “During the past 12 months, how many times did you travel away on holiday with your family?” with response options: “Not at all”; “Once”; “Twice”; and, “Three or more times”. Students who reported “Not at all” were classified as “No family holiday in last 12 months”.
Moved homes 2 or more times in the last 12 months	Moving frequently was assessed by the question: “In the last 12 months, how many times have you moved homes?” with response options: “I haven’t moved homes”; “I have moved once”; “I have moved two times”;”I have moved three or more times”. Students who responded two or more times were classified as “Moved homes 2 or more times in the last 12 months”.
Living room or garage used as bedrooms	Lack of bedrooms was assessed by the question: “What places are used as bedrooms in your home? (You can choose as many as you need)” with the “yes” or ‘no” response options to the following choices: “Living room”; “Garage”; “Caravan”; “Other rooms that aren’t bedrooms”; and, “None of these”. Students who reported a living room or garage used as bedrooms were classified as ‘Living room or garage used as bedrooms”.
No parent at home with full-time employment	Parental employment was explored through two questions, separately for mothers and fathers: “Does your dad (or someone who acts as your dad) have a job?”, with response options: “Yes – full time”; “Yes – part time”; “No”; ‘I don’t know”; and, ‘Does not apply to me”. Students were also asked who they lived with in their home, with response options including their mother and/ or father. Students who responded that both parents were not in fulltime employment or that the single parent they lived with was not full-time employment were classified as having “No parent at home with full-time employment”.

Neighbourhood deprivation was measured using the New Zealand 2013 Deprivation Index (NZDep2013). NZDep2013 assesses nine dimensions of neighbourhood deprivation (rates of no access to internet, unemployment, recipient of state-funded benefits, household income, home ownership, lack of educational qualifications, single parent families, overcrowding, and no access to a car) using 2013 New Zealand census data [22]. During the survey students were asked to provide their home address in order to ascertain the small area geographical unit or meshblock (unit of approximately 100 residents) in which they lived. Each participating student’s NZDep2013 was calculated by linking their residential meshblock number to their respective neighbourhood NZDep2013. The NZDep2013 were grouped into quintiles ranging from neighbourhoods with the low socioeconomic deprivation to neighbourhoods with high levels of socioeconomic deprivation.

Each student’s residential meshblock was also used to classify their area of residence as main urban (major urban areas with a minimum population of 10,000 people), minor urban (population between 1000 and 9999 people) and rural (populations less than 1000 people).

### Statistical analyses

Descriptive analyses compared the nine household deprivation indicators by demographic groups using chi-square tests of independence. Latent class analyses (LCA) were used to identify groups of students based on dichotomised indicators of household deprivation. The optimal number of classes is determined by estimating models with an increasing number of classes and comparing fit indices between models. Models were fitted using PROC LCA using pseudo-maximum likelihood estimation with robust standard errors to account for within-school correlation [23, 24]. Classification quality was assessed using recommended indices, including the Akaike information criterion (AIC), the Bayesian information criterion (BIC), and normalised entropy criterion [25]. Missing data were assumed to be ignorable and handled through full information maximum likelihood; 98.6 % of students had data for eight or more indicators. Only 23 students had missing data on all nine indicators and were excluded from further analyses. Participants were then assigned to their most likely group based on maximum probability assignment.

Odds ratios were used to estimate the relationship between the health indicators and the assigned deprivation groups from the LCA, controlling for age, sex, ethnicity, urban location and neighbourhood deprivation as covariates. Empty cross-classified multilevel models were used to explore the proportion of variance among the health and behaviour indicators at the school and neighbourhood levels. The portion of variance at the neighbourhood level for depressive symptoms, overweight/obese and cigarettes smoking over the total variance, including an assumed individual variance of *π*^*2*^*/3,* was 0.4, 0.6 and 2.3 %, respectively. The portion of variance at the school-level over the total variance for depressive symptoms, overweight/obese and cigarettes smoking was 0.8, 3.6 and 2.9 %, respectively.

Cross-classified multilevel logistic models with cross-level interactions between socioeconomic group and the level of socioeconomic deprivation of neighbourhoods were used explore the relationship between household socioeconomic deprivation and neighbourhood socioeconomic deprivation on the health indicators controlling for age, sex, ethnicity, and urban location. All analyses accounted for the sampling cluster design and unequal probabilities for selection. Estimation techniques used restricted pseudo-likelihood estimation using the GLIMMIX procedure in SAS version 9.3.

## Results

The most common indicator of household deprivation (Table 2) was “no family holiday in the last 12 months” (22 %), followed by “living room or garage used as bedroom” (16 %). The least common indicator was households having “no car” (2 %) or “no computer” (4 %). There were marked differences between ethnic groups, with Māori students and Pacific students being more likely to report all indicators of household deprivation than students from New Zealand European, Asian and other ethnic groups (*p* values all <0.001). Indicators of household deprivation were all consistently higher in less affluent neighbourhoods, with notably high proportions of household deprivation in the highest quintile of neighbourhood deprivation.

**Table 2:** Indicators of household deprivation among secondary schools students (*N* = 8500)

		N (%)	No car n (%)	No phone n (%)	No computer n (%)	Parents worry about having enough money for food n (%)	More than 2 people per bedroom n (%)	No family holiday in last 12 months n (%)	Moved homes 2 or more times in the last 12 months n (%)	Living room or garage used as bedrooms n (%)	No parent at home with full-time employment n (%)
	Total	8500 (100)	179 (2.1)	518 (6.1)	372 (4.4)	920 (11.5)	465 (5.5)	1867 (22.0)	623 (7.3)	1375 (16.2)	536 (6.7)
Sex											
	Female	4623 (54.3)	86 (1.9)	278 (6.1)	202 (4.4)	523 (12.1)	258 (5.6)	996 (21.6)	342 (7.4)	643 (13.9)	284 (6.5)
	Male	3874 (45.7)	93 (2.5)	239 (6.2)	169 (4.4)	396 (10.8)	207 (5.3)	871 (22.5)	280 (7.2)	731 (18.9)*	250 (6.8)
Age											
	13 years and under	1838 (21.6)	44 (2.4)	139 (7.6)	103 (5.7)	213 (12.7)	103 (5.6)	364 (19.9)	148 (8.0)	308 (16.9)	118 (6.7)
	14 years	1896 (22.3)	44 (2.4)	117 (6.2)	85 (4.5)	230 (12.7)	118 (6.2)	406 (21.4)	165 (8.7)	323 (17.0)	110 (6.1)
	15 years	1755 (20.7)	27 (1.9)	116 (6.6)	85 (4.8)	198 (12.0)	96 (5.5)	387 (22.1)	128 (7.3)	267 (15.3)	115 (6.9)
	16 years	1578 (18.6)	37 (2.4)	88 (5.6)	57 (3.6)	162 (10.8)	86 (5.5)	372 (23.5)	92 (5.9)	254 (16.2)	101 (6.8)
	17 years and over	1422 (16.8)	27 (1.9)*	56 (4.0)*	41 (2.9)	116 (8.6)*	62 (4.4)	335 (23.7)	89 (6.3)*	221 (15.5)	91 (6.9)
Ethnicity											
	Asian	1051 (12.43	29 (2.8)	16 (1.6)	8 (0.8)	80 (8.2)	47 (4.5)	218 (20.8)	43 (4.1)	180 (17.3)	71 (7.2)
	Māori	1701 (20.0)	52 (3.1)	211 (12.5)	116 (6.8)	228 (14.3)	99 (5.7)	378 (22.4)	178 (10.3)	374 (21.8)	148 (9.5)
	Other	511 (6.0)	7 (1.4)	9 (1.8)	11 (2.1)	31 (6.6)	19 (3.8)	135 (26.4)	36 (7.2)	54 (10.5)	36 (7.5)
	Pacific	1201 (14.3)	56 (4.8)	177 (15.0)	174 (14.8)	265 (24.0)	257 (21.5)	463 (38.7)	148 (12.4)	441 (36.9)	142 (13.0)
	NZ European	4024 (47.3)	35 (0.9)**	105 (2.6)**	63 (1.5)**	315 (8.2)**	41 (1.0)**	670(16.6)**	216 (5.4)**	324 (8.1)**	138 (3.5)**
Geographical Location											
	Main urban	6320 (74.7)	146 (2.3)	374 (6.0)	287 (4.6)	728 (12.3)	402 (6.4)	1407 (22.3)	448 (7.1)	1106 (17.6)	428 (7.2)
	Minor urban	946 (11.0)	25 (2.7)	92 (9.8)	50 (5.3)	112 (12.5)	31 (3.3)	220 (23.4)	100 (10.6)	137 (14.6)	68 (7.7)
	Rural	1234 (14.3)	8 (0.7)	52 (4.2)**	35 (2.9)*	80 (6.9)**	32 (2.6)**	240 (19.5)	75 (6.1) **	132 (10.7)**	40 (3.2)**
Neighbourhood deprivation											
	1 (low)	1684 (20.3)	6 (0.4)	17 (1.0)	10 (0.6)	77 (4.8)	16 (0.9)	209 (12.4)	80 (4.8)	121 (7.2)	32 (1.9)
	2	1576 (19.0)	8 (0.5)	39 (2.5)	29 (1.8)	109 (7.4)	29 (1.9)	277 (17.6)	78 (4.9)	134 (8.6)	64 (4.2)
	3	1574 (18.9)	25 (1.6)	66 (4.2)	35 (2.2)	161 (11.0)	50 (3.2)	333 (21.2)	109 (6.9)	202 (12.9)	73 (4.9)
	4	1515 (18.2)	36 (2.4)	108 (7.2)	70 (4.7)	188 (13.1)	70 (4.7)	400 (26.5)	119 (7.9)	282 (18.8)	95 (6.8)
	5	1975 (23.6)	100 (5.2)**	276 (14.2)**	219 (11.3)**	367 (20.0)**	285 (14.5)**	618 (31.5)**	219 (11.0)**	600 (30.35)**	261 (14.6)**

*p < 0.01, **p < 0.001

The AIC and adjusted BIC showed the greatest reduction between 2 and 3 class models with a more gradual reduction between 3 and 4 class models (Table 3). The BIC and consistent AIC show large improvements in fit between the 2 class and 3 class models and then increase in value between 3 class and 4 class models suggesting worsening fit. Based on these results a 3 class model was selected.

**Table 3:** Fit indices for Latent class models (2 to 5 classes)

	2 classes	3 classes	4 classes	5 classes	6 classes
Log-likelihood:	-20205.42	-20053.11	-20065.71	-20044.3	-20035.03
G-squared:	720.67	512.24	441.25	398.44	380.29
AIC:	758.67	570.24	519.25	496.44	498.29
BIC:	892.54	774.53	794.04	841.68	914
CAIC:	911.54	803.53	833.04	890.68	973
Adjusted BIC:	832.16	682.37	670.1	685.97	726.51
Entropy:	0.67	0.66	0.66	0.71	0.76
Degrees of freedom	492	482	472	462	452

*AIC* Akaike information criterion, *BIC* Bayesian information criterion, CAIC Bozdogan’s consistent AIC

**Fig. 1:**
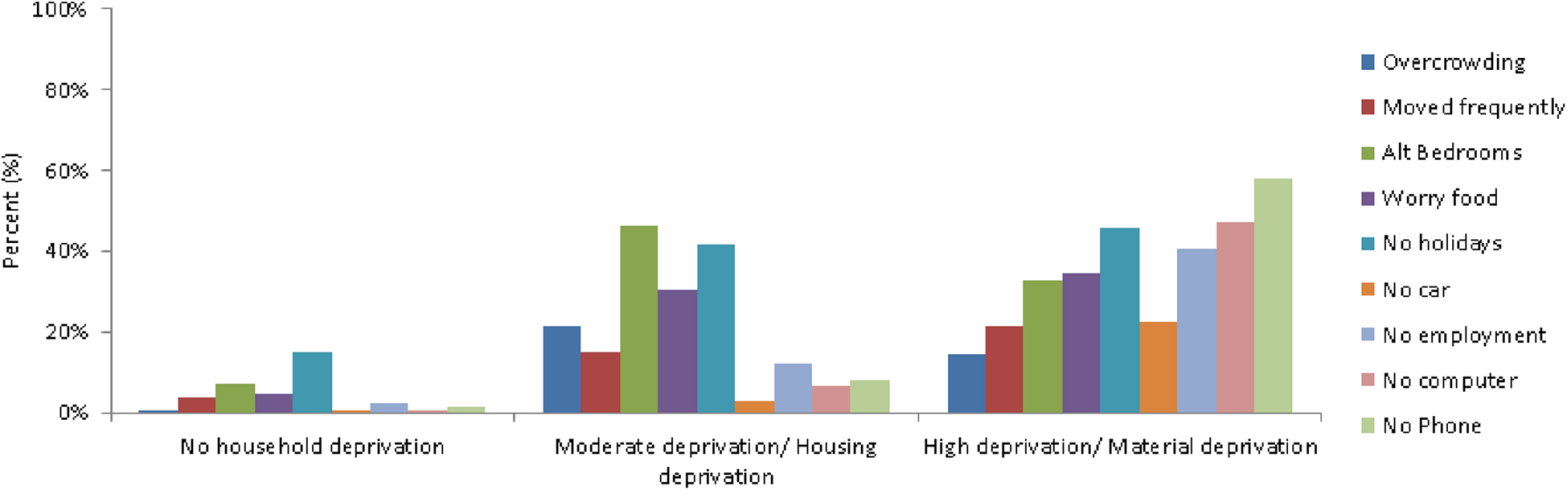
Prevalence of household deprivation indicators by deprivation group

Household deprivation response probabilities for the three groups based on latent class membership is shown in Fig. 1. A “No Household Deprivation” group was identified, with 67 % of students in this group reporting none of the indicators of household deprivation, and 31 % reporting one indicator of household deprivation. This group made up 80 % of the sample based on most likely class membership (Table 4). The second group identified had high levels of indicators suggesting housing stress such as alternative rooms used as bedrooms and overcrowding. This group also had high rates of students reporting that their parents worry about having enough money for food and no family holidays in the previous 12 months. In this ‘Housing Deprivation’ group, 58 % of students report two indicators of household deprivation, and 33 % of students in this group report three or more indicators of household deprivation. This group made up 15 % of students based on most likely group membership. The last group of students had high levels of household deprivation across all indicators, with 80 % of students reporting three or more indicators of household deprivation. This ‘Material Deprivation’ group made up 5 % of students based on most likely group membership. There were large ethnic group differences, with Pacific and then Māori students over represented in both deprivation groups.

**Table 4:** Demographics of deprivation groups and number of household deprivation indicators, n (%)

		No household deprivation	Housing deprivation	Material deprivation	*P* value
Total		6774 (80.0)	1301 (15.3)	397 (4.7)	
Sex					0.96
	Female	3695 (80.2)	696 (15.1)	215 (4.7)	
	Male	3078 (79.8)	604 (15.5)	181 (4.7)	
Age					0.02
	13 years and under	1436 (78.4)	285 (15.6)	109 (5.9)	
	14	1489 (78.9)	311 (16.3)	92 (4.9)	
	15	1404 (80.3)	261 (14.9)	84 (4.8)	
	16	1273 (81.0)	233 (14.8)	66 (4.2)	
	17 years and over	1163 (82.0)	211 (14.9)	44 (3.1)	
Ethnicity					<0.001
	Asian	892 (85.0)	144 (13.8)	13 (1.3)	
	Māori	1207 (71.9)	336 (19.8)	140 (8.2)	
	Other	440 (86.3)	61 (11.9)	9 (1.8)	
	Pacific	576 (48.1)	468 (39.1)	154 (12.9)	
	NZ European	3652 (90.9)	289 (7.1)	81 (2.0)	
Geographical Location					<0.001
	Main urban	4953 (78.5)	1059 (16.8)	299 (4.8)	
	Minor urban	740 (79.8)	125 (13.5)	62 (6.7)	
	Rural	1081 (88.2)	117 (9.1)	36 (2.7)	
Neighbourhood deprivation					<0.001
	1 (low)	1586 (94.2)	87 (5.1)	11 (0.7)	
	2	1422 (90.2)	126 (8.0)	28 (1.8)	
	3	1336 (85.1)	194 (12.3)	41 (2.6)	
	4	1187 (78.3)	257 (17.1)	69 (4.6)	
	5	1112 (57)	603 (30.7)	241 (12.3)	
Number of household deprivation indicators					
	None	4558 (67.3)	0	0	
	One	2104 (31.1)	108 (8.3)	0	
	Two	112 (1.6)	753 (57.8)	81 (20.2)	
	Three or more	0	440 (33.8)	316 (79.8)	

Students reporting “No Household Deprivation” had the lowest rates of overweight/ obesity (33 %), depressive symptoms (11 %), and weekly cigarette smoking (3 %) compared to students in the “Housing Deprivation” group (50, 21 and 8 %, respectively) and “Material Deprivation” group (54, 18 and 12 %, respectively). After adjusting for covariates (age, sex, ethnicity, geographical location and neighbourhood socioeconomic deprivation), students in the “Housing Deprivation” and “Material Deprivation” groups were 2.41 times the odds (95 % CI 2.22–2.62) and 1.84 times the odds (95 % CI 1.61–2.12), respectively, of being more likely to report high levels of depressive symptoms than students in the “No Household Deprivation” group. For cigarette smoking, students in the “Housing Deprivation” group were 2.21 (95 % CI 1.94–2.51) times the odds of being more likely to report weekly smoking compared with students from the No Deprivation group, and students in the “Material Deprivation” group were 2.97 (95 % CI 2.50–3.53) times the odds of being more likely to be report weekly smoking than students with “No Household Deprivation”.

There was a less clear relationship between overweight/obesity and household socioeconomic deprivation after adjusting for covariates. For example, the odds of overweight/obesity were only slightly higher among students in the “Material Deprivation” (aOR = 1.22, 95 % CI 1.09–1.34) and “Housing Deprivation” group (aOR =1.15, 95 % CI 1.08–1.23) compared to students with “No Household Deprivation” group.

**Fig. 2:**
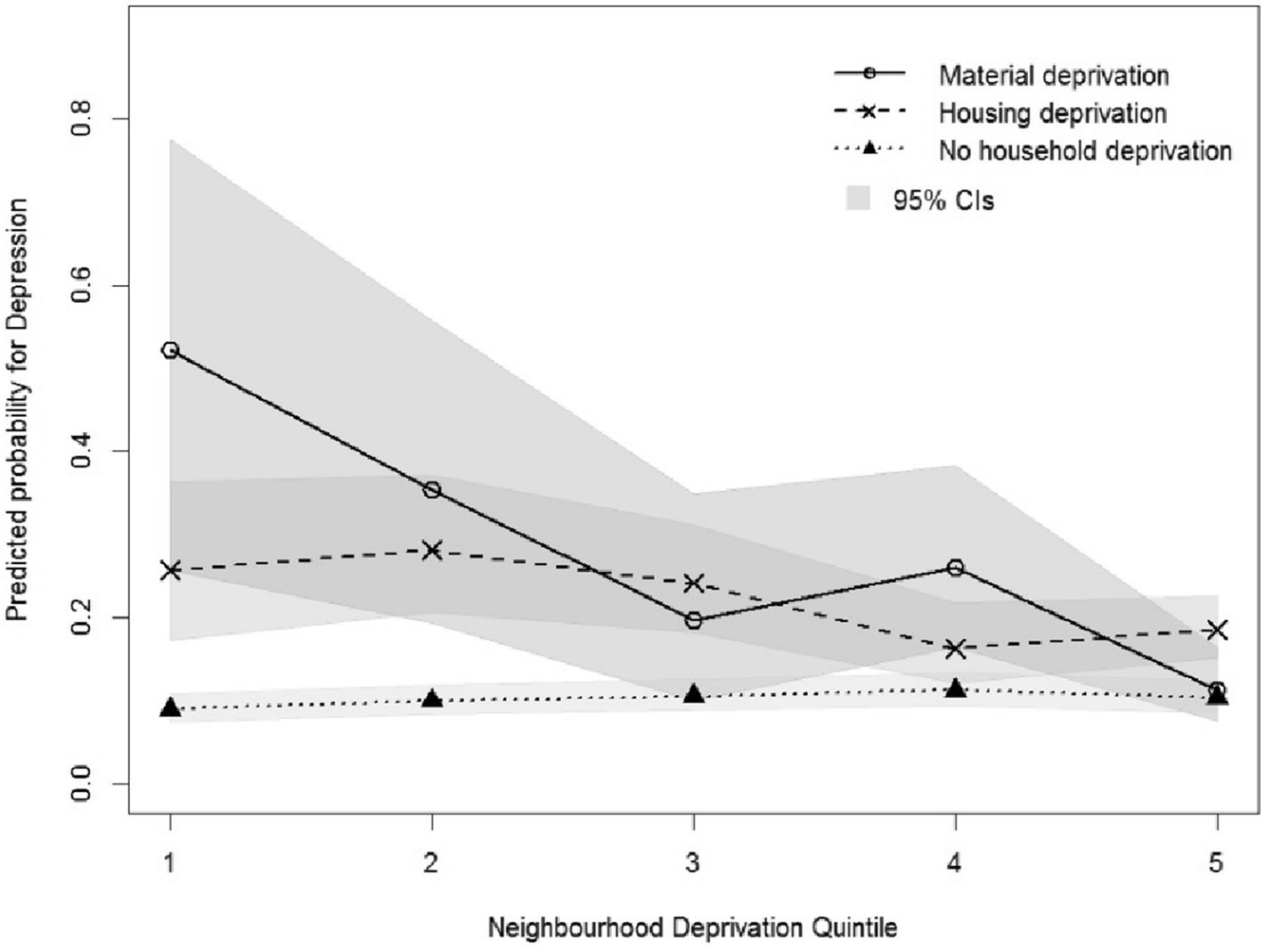
Predicted probabilities of depressive symptoms by household deprivation and neighbourhood socio-economic deprivation

**Fig. 3:**
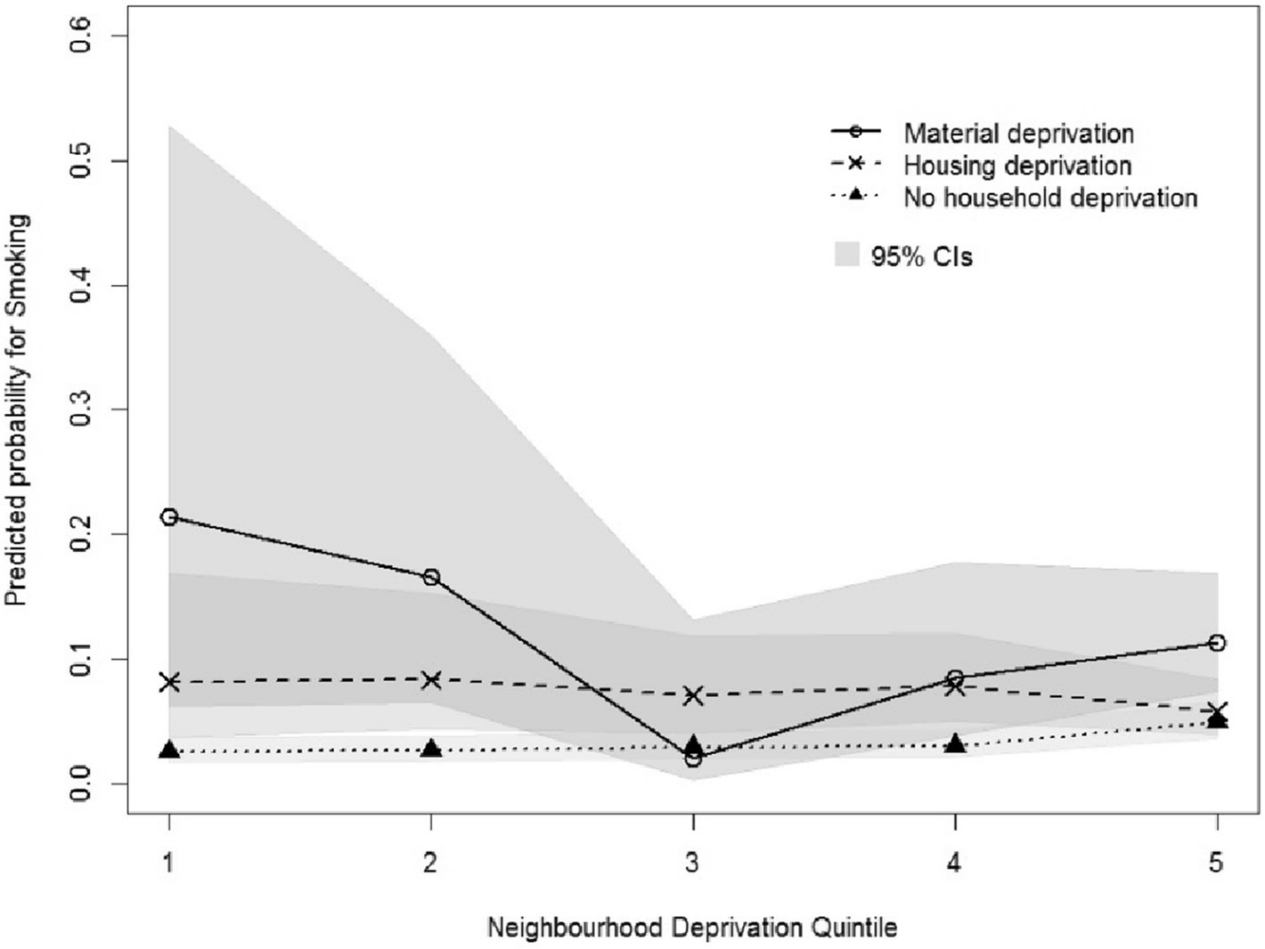
Predicted probabilities of cigarette smoking by household deprivation and neighbourhood socio-economic deprivation

There was a significant interaction between household deprivation and neighbourhood socioeconomic deprivation, with depressive symptoms (F = 3.65, *p* < 0.001); and weekly smoking (F = 2.12, *p* = 0.03); but not overweight/ obesity (F = 0.55, *p* = 0.82). Figures 2 and 3 show the estimates of the significant interactions for depressive symptoms and weekly smoking, respectively. Students experiencing household poverty were more likely to be at risk of depressive symptoms and report weekly cigarette smoking if they lived in more affluent neighbourhoods than if they lived in low (quintile 1) socioeconomic neighbourhoods. For students not experiencing household poverty there was no clear relationship between their risk of depressive symptoms or weekly cigarette smoking and level of socioeconomic deprivation of their neighbourhood.

## Discussion

In this nationally representative cross-sectional study, two patterns of household deprivation were found to be associated with poorer adolescent health indicators. One pattern showed high levels of housing stress along with moderate levels of material deprivation, with 15 % of adolescents in this group. The other deprivation pattern showed high levels across all indicators of family socioeconomic deprivation, particularly material deprivation such as no phone or computer at home, with 5 % of adolescents in this group. Adolescents living in households experiencing either pattern of socioeconomic deprivation were more likely to report significant depressive symptoms, weekly cigarette smoking and overweight/obesity. The overall alignment between measures of household deprivation and census-based estimates of neighbourhood deprivation supports the validity of this measure of poverty, and the presence of socioeconomic gradients for health indicators further highlights its usefulness as an indicator of household socioeconomic deprivation and a predictor of health indicators in young people.

We found almost one in five secondary school students in New Zealand live in households experiencing poverty. This is largely consistent with the proportion of children and adolescents living in low income households in New Zealand. Using household after-tax income adjusted for household size and composition and deducting housing costs, Perry [26] found that 20 % of adolescents aged 12 to 17 years are living in a household with an income level 60 % below the median income. However, it would be incorrect to assume we have identified the same group of adolescents living in poverty. There is a substantial mismatch between income thresholds and more direct measures of material hardship with typically less than 50 % overlap between the two measures. Both measures have their limitations. Low income thresholds may miss people with material deprivation and reasonable incomes but with extra expenses or costs due to illness or other factors. Material deprivation measures on the other hand, are necessarily subjective, can vary between contexts and over time and may fail to reflect lack of opportunities due to low income.

The large disparities in deprivation experienced by Māori and especially Pacific young people require more attention. Almost half of all Pacific students in this study are living in households experiencing poverty. This requires urgent action as the health impacts of socioeconomic deprivation during childhood and adolescence are considerable [27]. Māori and Pacific children and adolescents have historically experienced poorer health outcomes than other ethnic groups in New Zealand [28]. Our findings highlight the urgent need for policies to address these social and ethnic disparities.

This study was intended to show the development of a self-report measure of socioeconomic deprivation among young people. In particular, we were interested in how to combine individual and neighbourhood measures of deprivation. The cross-level interactions show that individual and neighbourhood measures of socioeconomic deprivation cannot be combined in a linear fashion. Researchers considering neighbourhood and individual measures of socioeconomic deprivation need to consider these two levels separately and take into account cross-level interactions.

This study found that risk of health and behaviour indicators such as depressive symptoms and cigarette smoking by adolescents experiencing poverty were worsened when living in more affluent neighbourhoods or attending more affluent schools. Previous mortality studies have shown that that poor people living in poor neighbourhoods have lower mortality than poor people living in more affluent neighbourhoods [29, 30]. There are several possible explanations of these findings. It may be due to perceived incongruity between an individual’s socioeconomic status and their peers causing psychosocial stress. This may be compounded by unequal access to social capital, particularly bonding social capital, whereby low socioeconomic individuals are socially excluded and become more isolated in more affluent neighbourhoods or schools. Adolescents may be particularly vulnerable to feelings of isolation and difference when they are unable to participate in activities in their communities and schools due to socioeconomic deprivation. This may then lead to rejection of conventional social norms with the adoption of cigarette smoking as a marker of difference [31].

Alternatively it may be a function of better support available to adolescents experiencing socioeconomic deprivation in low socioeconomic communities. In New Zealand, funding is targeted to low socioeconomic communities to help address socioeconomic disparities. This targeted funding includes both direct increases in funding to schools and the provision of health and social support services in these schools which have been shown to help address mental health concerns among students attending schools with these services [32]. Further studies would help elucidate if the observed interactions are from increased risk of more affluent environments or better support in more deprived communities.

### Limitations

There are a number of limitations to this study. It is well recognised that students who have dropped out of school come from lower socioeconomic backgrounds [33] and the omission of these students may have biased our findings in unexpected ways. There may be limitations in the range and specification of the nine indicators of socioeconomic deprivation which may have over- or under-estimated the true prevalence of socioeconomic deprivation. Further studies are needed to study the reliability and validity of these indicators for measuring socioeconomic deprivation among young people. We used a classify-analyse approach to examine the relationship between socioeconomic deprivation groups and distal health indicators, whereby individuals were assigned to their most likely group membership based on the maximum-probability assignment. As the true class membership is unknown, this approach does not take into account the uncertainty related to class membership. Lastly, given that this was a cross-sectional study we cannot be certain about the direction of our results. For example, it may be that students experiencing depression report their family socioeconomic situation more negatively. That said, this would not explain the findings in relation to overweight/obesity or cigarette smoking.

## Conclusions

We used latent class analyses to group secondary school students according to levels of household deprivation using a set of easily collected self-reported deprivation indicators. The analyses reveal two groups of household poverty, both were associated with poorer health indicators, especially among students experiencing household poverty in more affluent neighbourhoods and schools. Given the nationally representative and random sample of schools and students, these findings should be generalisable to the wider secondary school population in New Zealand. These findings demonstrate the importance of analysing socioeconomic deprivation at multiple levels and considering the complex interplay between an individual and their environment.

## Abbreviations

AIC: Akaike information criterion
BIC: Bayesian information criterion
HBSC: Health Behaviour in School-Aged Children
LCA: Latent class analyses
NZDep2013: New Zealand 2013 Deprivation Index
